# Deformation-Aware MR-TRUS Image Translation for Prostate Cancer Brachytherapy

**DOI:** 10.21203/rs.3.rs-7958252/v1

**Published:** 2025-11-18

**Authors:** Yunkui Pang, Xu Chen, Yunzhi Huang, Brenton Lian, Pew-Thian Yap, Jun Lian

**Affiliations:** 1 Department of Computer Science, University of North Carolina, Chapel Hill, NC 27599, USA; 2 College of Computer Science and Technology, Huaqiao University, Xiamen 361021, China; 3 School of Automation, Nanjing University of Information Science and Technology, Nanjing 210044, China; 4 Department of Radiology and Biomedical Research Imaging Center, University of North Carolina, Chapel Hill, NC 27599, USA; 5 Department of Radiation Oncology, University of North Carolina, Chapel Hill, NC 27599, USA

**Keywords:** MRI, TRUS, Image-to-Image Translation, Prostate Brachytherapy

## Abstract

Transrectal ultrasound (TRUS) is widely used in prostate cancer brachytherapy for real-time imaging and to guide radioactive seed implants. However, its low soft tissue contrast limits precise anatomical localization. In contrast, magnetic resonance imaging (MRI) offers superior soft-tissue contrast but exhibits different structural deformations and nonlinear intensity responses compared to TRUS, preventing its direct use in brachytherapy procedures. To achieve accurate seed localization and soft tissue visualization, it often relies on manual annotations on both images, which are labor-intensive and prone to errors. We propose a region-of-interest (ROI)-guided modality translation framework that synthesizes TRUS images from MRI using structural priors and intensity-aware normalization. By generating synthetic TRUS images that are modality-aligned with real TRUS images, our approach simplifies subsequent deformable registration. The method combines cross-domain image synthesis with anatomical constraints to ensure fidelity in both geometric and intensity representations. Evaluations on multiinstitutional datasets demonstrate significant improvements in synthetic image quality and MR-TRUS alignment. This work advances multimodal medical image translation and supports robust cross-modality signal registration, thereby facilitating image-guided therapies and communication-driven clinical systems.

## 1 Introduction

**Figure 1:**
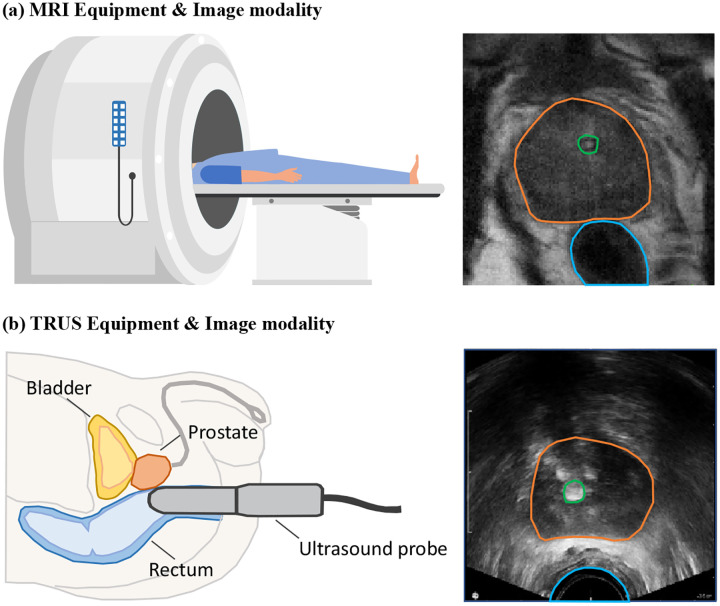
Imaging equipment and modality comparison for MRI and TRUS. (**a**) MRI system and a corresponding MR image. MRI images used in this study were acquired with an external coil, which does not cause organ deformation. (**b**) TRUS system with probe placement and corresponding ultrasound image. TRUS involves inserting an ultrasound probe into the rectum, which induces deformation. The prostate, urethra, and rectum are delineated in orange, green, and blue, respectively. Notable anatomical deformation is observed between the two modalities due to differences in acquisition techniques. Consequently, significant intensity discrepancies between modalities affect the appearance of structures such as the prostate and urethra.

Intermediate-risk prostate cancer is one of the most common malignancies in clinical practice. A prevalent and effective treatment approach for this condition is permanent low-dose rate (LDR) brachytherapy, an internal radiation therapy known for reliable tumor control [20, 23, 27]. As depicted in Fig. 1(a), magnetic resonance imaging (MRI) is routinely employed by physicians for lesion diagnosis due to its superior soft-tissue contrast. Despite these advantages, MRI is less commonly used directly for brachytherapy treatment planning due to its limited availability in operating environments. Consequently, transrectal ultrasound (TRUS), shown in Fig. 1(b), becomes the modality of choice for treatment planning and radioactive seed implantation. TRUS offers real-time imaging, ease of access, and cost-effectiveness, albeit at the expense of lower image quality than MRI. This disparity necessitates transferring lesion contours from MR to TRUS images to ensure accurate treatment planning and precise seed implantation. However, significant anatomical deformation and pronounced intensity differences between MRI and TRUS images pose critical challenges for accurately transferring contours using conventional contour-based deformable image registration (DIR).

**Figure 2:**
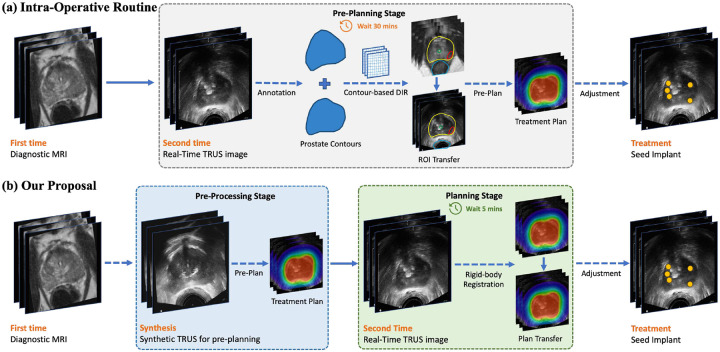
Comparison between the standard intraoperative treatment workflow and the proposed method. (**a**) The conventional approach requires manual intraoperative annotation of prostate contours to facilitate deformable image registration (DIR) for transferring contours from preoperative MRI to real-time TRUS images, significantly extending procedural time. (**b**) In contrast, our proposed method leverages preoperatively generated synthetic TRUS images to complete the majority of treatment planning in advance. This substantially reduces intraoperative annotation efforts, shortens the procedure duration, and streamlines the overall treatment workflow.

Clinical practice typically employs contour-based DIR methods to align these two imaging modalities and transfer contours, as illustrated in Fig. 2 (a). This approach, however, demands considerable additional effort from clinical personnel, typically consuming up to 30 minutes during operative procedures or necessitating an additional patient visit, thereby increasing overall treatment time and potentially causing procedural delays.

On observation that the central challenges in image-based deformable image registration (DIR) stem from significant modality differences and organ deformation, we propose an image-to-image translation framework based on deep learning that generates synthetic TRUS images from MR images and models organ deformation. This approach enables direct image-based DIR without requiring manual annotation, thereby significantly reducing intra-operative planning time before the implant procedure. As illustrated in Fig. 2(b), the new pre-planning workflow is streamlined into two sub-processes: (1) an image preprocessing stage that translates MR intensities into a TRUS-like appearance while simulating the anatomical deformations caused by TRUS probe insertion, and (2) a simplified image registration stage employing rigid body alignment to transfer the pre-made treatment plan from the synthetic TRUS image to real-time TRUS images. This pipeline reduces the intra-operative planning time from approximately 30 minutes to 5 minutes and eliminates the need for manual contouring on TRUS images.

Given the critical role of image-to-image translation in this workflow, it is essential to consider existing GAN-based synthesis methods and their limitations in handling modality differences and anatomical deformation. The application of generative adversarial networks (GANs) [5] to image-to-image translation has led to substantial advances in cross-modality medical image synthesis. Notably, CycleGAN [34] introduced the concept of cycle consistency, using two inverse generators to improve the fidelity of synthesized images. However, in our experiments, the method exhibits suboptimal performance due to the joint burden of style translation and content preservation, which becomes especially challenging in the presence of large anatomical deformations. In particular, the cycle consistency loss in CycleGAN struggles to accommodate non-rigid deformations between MR and TRUS images. To address this limitation, DCLGAN [8] replaces the cycle consistency loss with a dual-path synthesis framework and introduces a patch-wise noise contrastive loss to improve feature discrimination and reduce memory usage. Inspired by this design, our method incorporates a similar contrastive learning strategy to preserve anatomical structures and enhance modality adaptation while maintaining computational efficiency.

While image-to-image translation techniques have been widely explored in medical imaging tasks such as MR-to-CT and CBCT-to-CT synthesis [2, 19, 32, 33], there remains limited research focused on synthesis between MR and TRUS modalities. Existing MR-to-US synthesis methods are not readily applicable to MR-to-TRUS synthesis because they lack sufficient modeling of realistic anatomical deformations, particularly those induced by ultrasound probe insertion during TRUS acquisition. For instance, Reaungamornrat et al. [28] employed intensity-insensitive structural constraints and feature disentanglement for cross-modality image translation. However, their framework does not explicitly model the prostate-specific deformation caused by the transducer, which is critical in the context of prostate low-dose-rate (LDR) brachytherapy. Similarly, Jiao et al. [12] utilized a CycleGAN-based architecture to synthesize fetal brain MR images from ultrasound. Despite its success in fetal imaging, the method fails to produce clinically viable prostate images, as evidenced by our evaluations, due to inadequate anatomical fidelity and deformation modeling. Pang et al. [25] proposed a weakly supervised approach for prostate MR-TRUS synthesis that achieves improved performance. However, it relies on manual prostate contour annotations for all training TRUS images and omits the rectum, an organ-at-risk that must be spared during LDR brachytherapy. These limitations highlight the need for a dedicated MR-TRUS synthesis framework that robustly models organ deformation, eliminates dependence on manual annotations, and incorporates essential anatomical structures, such as the rectum, to ensure clinical applicability.

To address the aforementioned challenges, we propose a novel unpaired image-to-image translation framework specifically tailored for LDR prostate brachytherapy. Our method synthesizes TRUS-like images from MR inputs using unpaired training data, while effectively modeling the significant anatomical deformation between modalities. To regularize the synthesis process, we introduce a dual-level constraint mechanism combining both feature-level and image-level supervision. The feature-level constraints ensure modality translation fidelity by preserving global appearance and anatomical structure consistency.

In parallel, the image-level constraints guide the network to infer plausible shapes and spatial positioning of key organs, particularly the prostate and rectum, accounting for deformation induced by TRUS probe insertion. A key component of our strategy is the use of a Region-of-Interest (ROI) mask to enhance image-level supervision. This mask emphasizes critical anatomical regions such as the rectum and prostate and can be generated automatically, without requiring manual contouring, thereby enhancing the method’s practicality. Given the inherent ambiguity of deformation in unpaired data settings, this ROI-guided dual-level regularization significantly improves synthesis quality in clinically important regions. To further address the substantial intensity differences between MR and TRUS modalities, we introduce intensity-aware normalization. This module scales feature maps from both modalities to a common range, aligning feature vectors of similar anatomical structures to enhance their similarity in the latent space. This alignment improves translation accuracy and structure preservation. Our method demonstrates considerable potential for improving treatment efficiency. Enabling direct dose planning on synthesized TRUS images eliminates the need for time-intensive manual planning during intraoperative procedures. Furthermore, the synthetic images can be readily registered with intraoperative TRUS scans, facilitating real-time guidance for seed implantation.

## 2 Results

### 2.1 Architecture and System Design of Deformation-Aware MR-TRUS Image Translation Network

**Figure 3:**
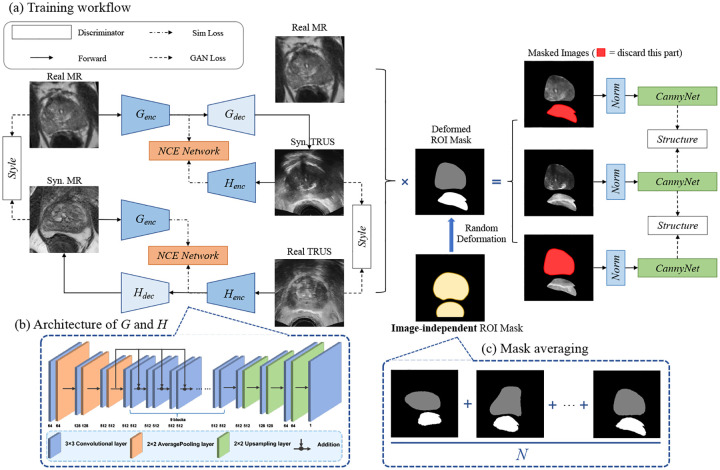
Training workflow and details of network design. (**a**) We use Generator G to synthesize TRUS images from the MR domain. We employ Generator H to produce Synthetic MR with TRUS images as input. The NCE Network takes feature maps from different domains for contrastive learning. We introduce an image-independent ROI mask and utilize a Canny network for MR anatomical structure consistency. (**b**) The architecture of the generators G and H. (**c**) The image-independent ROI mask $c_{0}$ we used in practice is produced by an average of manually annotated contours on TRUS images. We use this mask during training with random elastic deformation applied as a perturbation for each iteration.

As illustrated in Fig. 3, our Deformation-Aware MR-TRUS Image Translation network is formulated as an unpaired image-to-image translation task. Following the CycleGAN framework, the network employs two convolutional generators, G and H, responsible for bidirectional translation between MR and TRUS domains. Specifically, the generator G synthesizes TRUS-like images $\overset{ˆ}{y}$ from MR inputs x via $G(x)=\overset{ˆ}{y}$, while the generator H reconstructs MR images $\overset{ˆ}{x}$ from TRUS inputs y as $H(y)=\overset{ˆ}{x}$ to enforce consistency and enhance image quality. Both G and H share an identical architecture, as shown in Fig. 3, comprising three downsampling blocks, nine residual blocks [9], and three upsampling blocks. Operating on 2D slices extracted from volumetric data, the design achieves an optimal balance between anatomical fidelity and computational efficiency, making it scalable for large-scale clinical datasets.

To address the substantial anatomical deformation between MR and TRUS images, we discard the conventional cycle consistency loss and adopt a contrastive learning strategy inspired by [8, 26]. This approach enhances the model’s ability to extract deformation-aware features and ensures structural alignment across modalities. The framework incorporates a Noisy Contrastive Estimation (NCE) module, which learns to align feature representations by maximizing mutual information between input and synthesized images [24]. Positive samples are drawn from corresponding regions of the input and translated images, while negative samples are constructed from unrelated feature regions. This contrastive formulation guides the network to retain critical anatomical structures and improve modality translation. The NCE loss is formally defined as:

(1) $$L_{\text{NCE}}=-\text{log}\left(\frac{\text{exp}\left(\text{sim}\left(v,v^{+}\right)\right)}{\text{exp}\left(\text{sim}\left(v,v^{+}\right)\right)+\sum_{n=1}^{N}\text{exp}\left(\text{sim}\left(v,v^{-}\right)\right)}\right)$$

where $\text{sim}(u,v)=u^{T}v/\|u\|\|v\|$ quantifies cosine similarity [6].

#### 2.1.1 Region-of-Interest Structure Guidance

The unpaired TRUS image during training cannot impose a strict structural constraint, leading the network to generate anatomically plausible but inaccurate prostate representations and handle rectum deformation inconsistently. To mitigate this, we constrain the loss computation within an ROI mask, ensuring the model preserves prostate structures from input MR images while learning realistic rectum deformations from TRUS images. The ROI mask $c_{0}$ is derived from the average of pre-annotated TRUS volumes, providing a coarse but consistent localization for synthesis guidance. This mask directs the network to focus on structurally preserving the prostate while capturing realistic rectal deformation, improving anatomical coherence across synthesized images.

However, because $c_{0}$ represents an averaged anatomical approximation, it does not align precisely with individual cases, resulting in partial coverage of each image. Given inter-patient variations in prostate and rectum size and positioning, a fixed ROI mask may limit the ability of the network to generate anatomically accurate rectal deformations while maintaining prostate integrity. Inspired by [15], which augments noisy images to match predefined shapes for improved shape representation learning, we adopt an inverse strategy: introducing controlled perturbations to the ROI mask to encourage the model to generalize to natural MRI-to-TRUS deformations and patient-specific variations without enforcing strict region coverage. Additionally, the perturbation assists the model to retain anatomical structures from the input while avoiding overfitting to unrelated features. Additionally, this design ensures robustness without requiring precisely aligned annotations. To implement this, we apply random elastic deformations [29] to the initial ROI mask $c_{0}$ during training. The randomly deformed mask $c_{i}$ for each iteration is defined as:

(2) $$E\left(c_{i}\right)={𝒢}_{\sigma }\cdot ℛ\left(c_{0}\right)$$

where ${𝒢}_{\sigma }$ is a Gaussian smoothing filter with standard deviation σ that controls the deformation magnitude. ℛ is the is a randomly sampled displacement field applied to $c_{0}$. For training, elastic deformations with a 3 × 3 grid and a standard deviation of σ=20 are applied.

To enforce anatomically accurate structure representation, we impose constraints using edge-based adversarial losses. Edge information is extracted from the input MR, reference TRUS, and synthesized TRUS images using the Sobel filter [14]. These edges are then filtered to retain only those within the ROI mask, ensuring that the adversarial loss targets the most relevant anatomical regions.

Unlike L1 or MSE loss, which enforces direct pixel-wise consistency, adversarial loss allows the model to generalize to the natural deformation of the prostate and rectum from MRI to TRUS. Since we employ unpaired data for training, strict structural consistency with MRI can lead to artifacts such as duplicated rectal structures or unrealistic overlapping with the prostate in the synthesized TRUS images. By adopting an adversarial approach, we encourage the model to synthesize anatomically plausible structures that align with MR modality while adapting to modality-specific deformations.

To ensure the synthetic images accurately preserve the anatomical structures of the prostate in MR images, while realistically synthesizing the rectum with deformations consistent with TRUS images, we apply adversarial losses separately to the prostate and rectum regions. Specifically, the adversarial loss for the prostate enforces edge similarity of anatomical structures between synthetic and MR images:

(3) $$L_{\text{p}}=E_{p\sim P}\;\text{log}\;D_{P}\left(c_{\text{p}}\cdot 𝒮(x)\right)+E_{p\sim P}\;\text{log}\left(1-D_{P}\left(c_{\text{p}}\cdot 𝒮(G(x))\right)\right)$$

where $c_{\text{p}}$ represents the estimated prostate contour, 𝒮(x) denotes the extracted edge map, and $D_{P}$ is the discriminator for the prostate region. Similarly, the adversarial loss for the rectum constraints the edge similarity of rectum between synthetic images and TRUS images:

(4) $$L_{\text{r}}=E_{r\sim R}\;\text{log}\;D_{R}\left(c_{\text{r}}\cdot 𝒮\left(x\right)\right)+E_{r\sim R}\;\text{log}\left(1-D_{R}\left(c_{\text{r}}\cdot 𝒮(G(x))\right)\right)$$

where $c_{\text{r}}$ represents the estimated rectum contour. The loss function is illustrated in Fig. 3 as structure loss.

#### 2.1.2 Intensity-Aware Normalization

MR images typically exhibit higher contrast and uniformity, whereas TRUS images are noisier and have lower contrast, making direct synthesis difficult. To address this, we introduce intensity-aware normalization, which aligns MR feature maps to match the intensity distribution of TRUS images, thereby improving synthesis consistency.

By incorporating intensity-aware normalization with ROI-guided constraints, the transformation is focused on clinically relevant regions, such as the prostate and rectum, ensuring anatomically plausible outputs. The normalization process standardizes the feature maps based on the mean (μ) and standard deviation (σ) of the TRUS domain:

(5) $${\text{Norm}}_{x}=\sigma (y)\cdot \frac{x-\mu (x)}{\sigma (x)}+\mu (y),$$

where x and y represent MR and TRUS feature maps, respectively. Similarly, for the TRUS-to-MR synthesis branch, the normalization is defined as:

(6) $${\text{Norm}}_{y}=\sigma (x)\cdot \frac{y-\mu (y)}{\sigma (y)}+\mu (x)$$

#### 2.1.3 Optimization Objective Summary

The overall training objective integrates multiple loss terms to guide the two generators, G and H, in synthesizing anatomically accurate and visually coherent synthetic TRUS images. Following CUT [26], we employ adversarial loss $L_{\text{adv}}$ to ensure realistic synthesis by distinguishing between real and synthesized images, and identity loss $L_{\text{idt}}$ to maintain domain-specific intensity characteristics. Additionally, the contrastive learning loss $L_{\text{nce}}$ (Eq.3, 4) enforce structural consistency, while the style loss $L_{\text{style}}$ (Eq. 7) further refines local intensity distributions.

The loss function for generator H is formulated as:

(7) $$L_{\text{H}}=\lambda_{1}\cdot L_{\text{adv}}(H(y),x)+\lambda_{2}\cdot L_{\text{nce}}(H(y),y)+\lambda_{3}\cdot L_{\text{idt}}(H(y),x)$$

Similarly, for generator G, the loss function is:

(8) $$L_{\text{G}}=\lambda_{4}\cdot L_{\text{adv}}(G(x),y)+\lambda_{5}\cdot L_{\text{nce}}(G(x),x)+\lambda_{6}\cdot L_{\text{idt}}(G(x),y)+\lambda_{7}\cdot \left(L_{\text{p}}+L_{\text{r}}\right)$$

Each loss component is weighted by empirically determined parameters: $\lambda_{1}=0.5,\lambda_{2}=\lambda_{3}=0.25,\lambda_{4}=0.5,\lambda_{5}=\lambda_{6}=0.25$, and $\lambda_{7}=0.01$. The weighting parameters (λ values) were initially determined empirically through systematic experiments conducted on our training dataset. To ensure stability and generalizability, we evaluated multiple configurations of λ values and selected those that consistently balanced synthesis quality and anatomical accuracy across validation cases.

### 2.2 Experiment Design

We have devised three distinct tasks to assess both the performance and clinical viability of our proposed method: 1) An image quality comparison with images synthesized by existing state-of-the-art methods. 2) A registration quality comparison to evaluate whether the synthesized image can mitigate registration difficulty and replace real TRUS in clinical applications. 3) Clinical evaluation to assess treatment planning on synthetic TRUS images and registration results for landmarks and lesions. 4) an ablation study to illustrate the significance and contribution of each component.

We use the pre-trained registration-based method Hu et al. [10] as the baseline. As for the comparison models, we select six state-of-the-art modality translation methods: CycleGAN [34], ImgSA [13], UNIT [21], MUNIT [11], DRIT++ [17], DCLGAN [8], and Chen et al. [3]. Besides, we also include the most related work [25] (denoted as Weakly MRUS) proposed for prostate MR-to-US image translation. In contrast to the dataset utilized in [25], which solely emphasizes the synthesis quality of the prostate, our novel dataset encompasses the comprehensive view of both the prostate and rectum, thereby augmenting the complexity of the task. We run our experiments on an NVIDIA Titan XP GPU.

### 2.3 Dataset Preparation

We collected a total of 2114 intra-operative TRUS images for treatment planning and 3366 MR images from the UNC hospital, NCI-ISBI 2013 [1], and I2CVB [18] dataset. The TRUS images were acquired with an endorectal probe, and the MR images were acquired with an external coil. We select 1360 TRUS images and 2312 MR images for training, 68 paired TRUS and MR images for validation, and 1020 paired images for testing.

We resample all the images to 0.3 × 0.3 × 1 mm^3^. With the use of NiftyReg [22], we rigidly registered MR and TRUS images to their templates separately. We then cropped each image to a size of 296 × 296 × 34 after registration as a trade-off between memory efficiency and image resolution. To standardize data processing and facilitate model training, we apply the affine transformation to all MRI volumes to a uniform size using a selected template volume. This optimizes data storage and computational efficiency. During testing, the synthetic TRUS images are restored to their original field of view for evaluation, ensuring that the performance of the model is assessed in a clinically relevant context. We apply image volume normalization, scaling them to the interval of [−1, 1], prior to inputting them into the models.

### 2.4 Evaluation Metrics

To assess the quality of synthesized TRUS images, we employ multiple evaluation metrics. Peak Signal-to-Noise Ratio (PSNR) and Structural Similarity Index Measure (SSIM) are used to quantify the similarity between synthesized and real TRUS images, with results summarized in Table 1. To further evaluate the realism of the synthesized images, we use Fréchet Inception Distance (FID) and Normalized Cross-Correlation (NCC). While FID was originally developed for natural image assessment and its use in medical imaging has been debated [30], prior studies [4, 7, 16, 31] have demonstrated its relevance in GAN-based medical image synthesis. In our case, we validated the FID score against human rankings of image similarity and found it to be a reliable indicator of perceived image quality.

To evaluate registration accuracy, we compute the Dice Similarity Coefficient (DSC) between prostate contours registered from synthesized TRUS images and those from real TRUS images. Registration is performed by first aligning the synthesized image to the real TRUS image to obtain a transformation matrix, which is then applied to the prostate contour originally delineated on the MR image. The DSC is computed between the transformed MR prostate contour and the corresponding TRUS annotation.

We also computed the Target Registration Error (TRE) for key anatomical structures, including the prostate, urethra, and dominant intraprostatic lesion, to validate the anatomical consistency of the synthetic images. The quantitative results are reported in Section 3.

For ROI-specific evaluation in Table 2, PSNR is calculated using only pixel values within the ROI mask, while SSIM is computed within a bounding box tightly enclosing the ROI.

## 3 Clinical Evaluation

To evaluate the feasibility of using synthetic ultrasound images for treatment planning, we conducted a study on ten prostate cancer patients. For each patient, the prostate was contoured on the synthetic ultrasound image and registered to the corresponding clinical ultrasound image by maximizing prostate volume overlap. The radioactive seed positions from the clinical treatment plan were projected onto the synthetic images, and the dose distribution was recalculated to generate a research plan.

To assess dosimetric consistency, we compared the percentage of prostate volume receiving 100% of the prescription dose (V100%) between the clinical and research plans (Fig. 8). The clinical plans yielded a mean V100% of **96.68** ± **2.42**%, while the research plans based on synthetic images resulted in a V100% of **95.41** ± **3.02%**. The difference was not statistically significant using the conventional clinical standard of 5% of the difference in target coverage, indicating that treatment planning using synthetic ultrasound images closely aligns with conventional clinical planning. This suggests that synthetic images generated by our method may serve as a reliable alternative to real ultrasound images, potentially reducing the need for additional imaging and expediting clinical workflow.

To further validate the anatomical consistency of the synthetic images, we computed the Target Registration Error (TRE) for key anatomical structures, including the prostate, urethra, and dominant intraprostatic lesion. TRE was measured as the Euclidean distance between the centroids of the manually segmented structures in the real TRUS images and the corresponding contours transferred from synthetic TRUS images via rigid-body registration. The results showed a mean TRE of **2.239** ± **1.02 mm** for prostate centers, **1.023** ± **0.84 mm** for urethra centers, and **0.794** ± **0.44 mm** for lesion centers. These values indicate a high degree of spatial alignment, further supporting the clinical applicability of synthetic TRUS images in treatment planning.

### 3.1 Qualitative and Quantitative Evaluation

**Figure 4:**
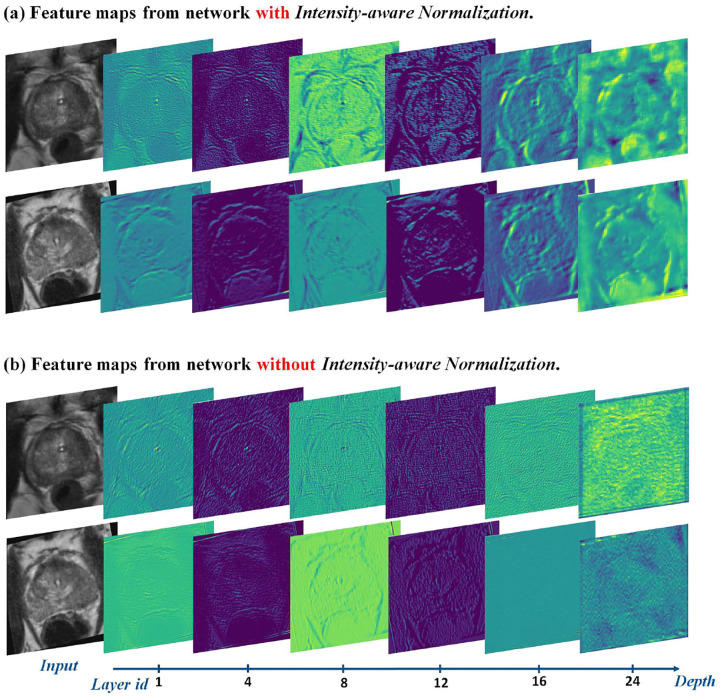
Feature maps produced by trained networks with/without intensity normalization. Networks with intensity-aware normalization can pass more information to the deeper layers in the model.

**Figure 5:**
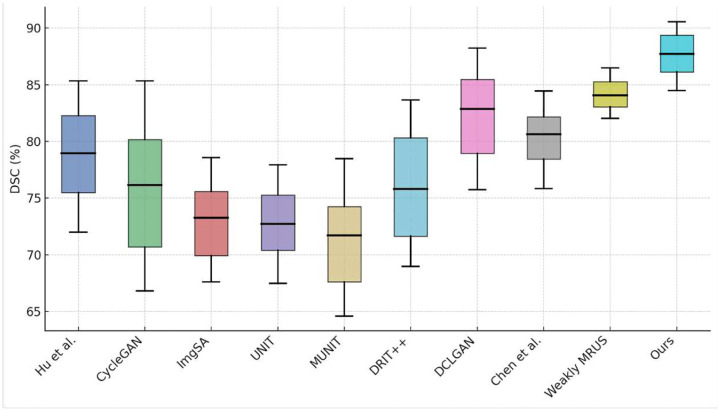
Box plot of Dice Similarity Coefficient (DSC) scores across different image translation methods for MR-to-TRUS synthesis.

**Figure 7:**
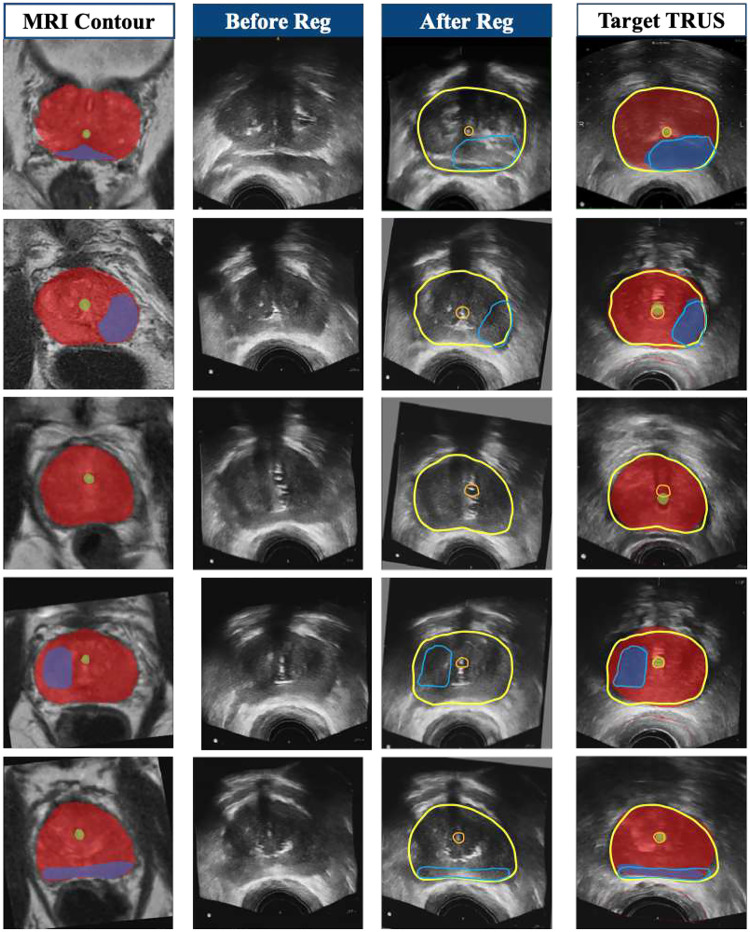
Rigid-body registration results using images synthesized by our method. The region-filled contours (red for prostate, green for urethra, and blue for dominant intraprostatic lesions) in the MR and TRUS images were manually annotated by clinical experts. Since synthetic TRUS images are generated without deformation, MR contours can be directly transferred to the corresponding synthetic TRUS images without modification. The column labeled ‘Before Reg’ shows images prior to registration, while ‘After Reg’ displays the results after rigid-body registration to the target TRUS image. The boundary-only contours (yellow for prostate, orange for urethra, and light blue for dominant intraprostatic lesions) represent these MR-derived annotations after being mapped to real TRUS images via rigid-body registration between synthetic and real TRUS images. These boundary-only contours were then overlaid onto the real TRUS images to visualize alignment and overlapping regions. Target Registration Error (TRE) was computed between the centroid positions of the transferred boundary-only contours and the corresponding ground-truth region-filled contours on real TRUS images. The TRE values are as follows: 2.239 ± 1.02 mm for prostate centers, 1.023 ± 0.84 mm for urethra centers, and 0.794 ± 0.44 mm for lesion centers.

**Figure 8:**
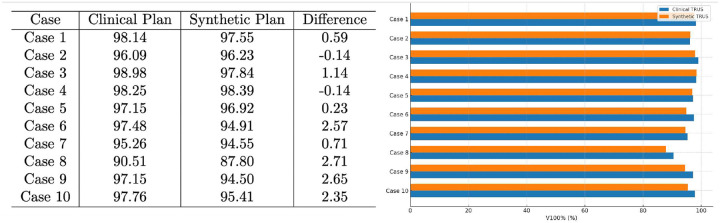
Comparison of V100% between treatment plans performed on real-time clinical TRUS images and synthetic TRUS images. 5% of target coverage difference is commonly used to indicate clinical significance.

We will first conclude the qualitative results of seven state-of-the-art methods and our method, as shown in Fig. 4. Overall, it is evident that the images synthesized by our method have a style closer to the authentic TRUS images. The image generated by DCLGAN and Weakly MRUS is also of high quality. Nevertheless, the prostate and rectum details synthesized by these two models are not satisfying. Further, the images produced by our model have more vivid prostates and reasonable rectums. The anatomical structures from the MR images are well preserved, while the rectum is correctly deformed. Fig. 5, Table 1 and Table 2 report the quantitative performance. The pre-trained baseline method [10] did not perform well because we do not have enough paired and annotated MR-TRUS images to fine-tune the model. Our method achieves the best or near-best results on all the metrics. Note that our method outperforms the comparison methods by a large margin regarding FID. These findings indicate that the style of our synthetic image demonstrates a more remarkable resemblance to the ground truth compared to alternative models. Furthermore, the high DSC score achieved by our model suggests that the prostate structure is well preserved from the MR image, and the boundary is vivid enough for high-quality registration. Fig. 7 presents the registration results on the prostate, urethra, and lesions. We perform paired t-tests to compare our method against existing approaches. The statistical results demonstrate that our method significantly outperforms the alternatives.

### 3.2 Ablation Study

**Figure 9:**
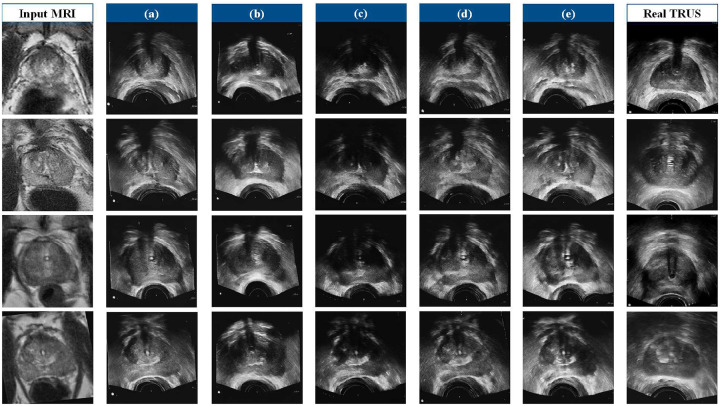
Visualization of the ablation study. (a): *w/o. cycle breaking*, (b): *w/o. NCE network*, (c): *w/o. normalization*, (d): *w/o. structure constraint,* and (e): *w/o. style adjustment*.

We investigate the effectiveness and significance of each component from the ablation study. We train five variants of our model by changing one component at a time: (a) add the cycle consistency loss (*w/o. cycle breaking, ”cycle breaking” means to avoid cycle consistency loss*), (b) drop the NCE network (*w/o. NCE network*), (c) drop the intensity-aware normalization (*w/o. normalization*), (d) drop the ROI-guided anatomical structure constraint (*w/o. structure constraint*), and (e) drop the style adjustment (*w/o. style adjustment*). The qualitative and quantitative results are shown in Fig. 9 and Table. 3. The results show that the style adjustment only affects the appearance (PSNR and FID) of the synthesized image. The NCE network, intensity-aware normalization, and the anatomical structure constraint influence the structure translation quality in the synthesized image. The intensity-aware normalization seems to have the most significant influence on image quality. The anatomical structure constraint is not redundant. Without it, the network will tend to generate artifacts in the prostate and rectum.

To evaluate the robustness of the Canny edge filter in our framework, we conducted an experiment assessing the impact of different edge detection thresholds on synthesis quality. During training, the images are scaled to the range of [0, 255], and edges are extracted using the Canny filter with an initial threshold of 300. The extracted edges are then normalized to the range of [−1, 1] before being incorporated into the loss computation. To examine the sensitivity of this threshold, we tested five values (200, 250, 300, 350, 400) and computed PSNR and SSIM scores, as shown in Table 4. The results indicate that synthesis quality remains relatively stable across different thresholds, with only a slight decline in performance at the lowest (200) and highest (400) values. This suggests that the edge-based structural constraint is robust to minor variations in threshold selection, further reinforcing its reliability in guiding anatomically coherent synthesis.

## 4 Conclusion

We introduced an innovative ROI-guided method for synthesizing MR-TRUS images using unpaired data. Starting from CycleGAN, we explored techniques to enhance model performance, forming a roadmap. Our intensity-aware normalization NCE network-based approach showed significant performance improvements. Furthermore, this method (without ROI-guided anatomical structure constraint) is versatile for other cross-modality image translations. Experimental results demonstrate its ability to generate vivid synthetic TRUS images, facilitating easier registration with existing tools. Additionally, the synthesized image reduces obstacles in MR-TRUS registration, suggesting potential applicability in prostate cancer brachytherapy.

## Figures and Tables

**Figure 6:**
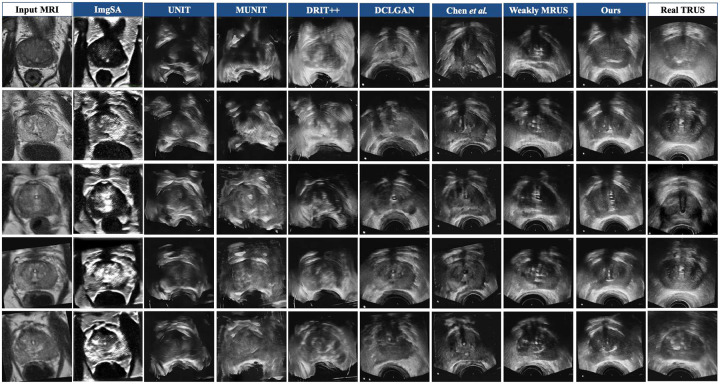
The qualitative outcomes of our proposed model, as well as other image-to-image translation techniques renowned for their success in natural images.

**Figure 10:**
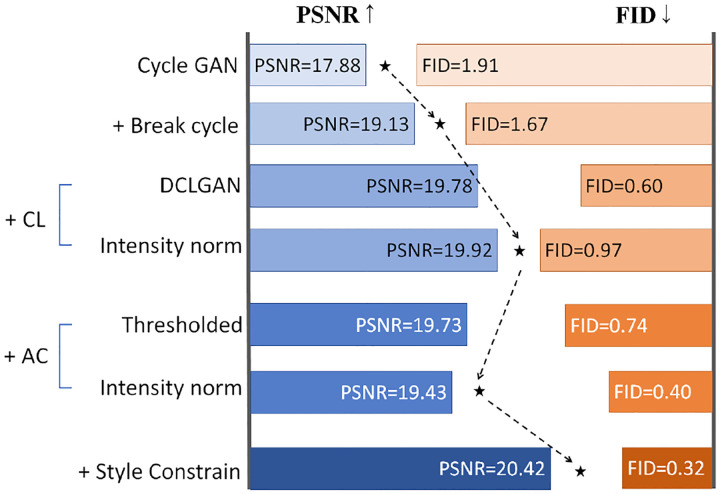
Exploration roadmap showcasing the progression from CycleGAN to our final proposed method. Performance evaluation of each modification is based on PSNR (higher is better) and FID (lower is better). ”CL” denotes contrastive learning, with two different approaches investigated. ”AC” represents anatomical structure constraint, explored through two constraint methods. Lastly, style adjustment was employed to enhance PSNR scores.

**Table 1:** Quantitative evaluation results. The pre-trained registration-based method Hu et al. [10] is used as the baseline. We employ several metrics to assess the overall image quality between the synthesized and target TRUS images. These metrics include PSNR, SSIM, FID (Fréchet Inception Distance), and NCC (Normalized Cross-Correlation). Higher values indicate better quality across all metrics except for the FID score. Additionally, we utilize the Dice Similarity Coefficient (DSC) to measure the registration difficulty between the synthesized image and the target TRUS image. The p-values are reported in parentheses.

Method	PSNR ↑	SSIM ↑	FID ↓	NCC ↑	DSC (%) ↑
Hu et al.	-	-	-	-	78.65 ± 6.74
Cycle GAN	17.08 ± 0.71 (8*e*^−15^)	0.41 ± 0.03 (4*e*^−7^)	1.91 ± 0.65 (2*e*^−18^)	0.72 ± 0.02 (1*e*^−23^)	76.01 ± 9.39 (3*e*^−5^)
ImgSA	17.76 ± 0.83 (1*e*^−11^)	0.38 ± 0.03 (3*e*^−10^)	5.01 ± 0.39 (3*e*^−52^)	0.53 ± 0.03 (2*e*^−45^)	73.14 ± 5.57 (1*e*^−8^)
UNIT	18.26 ± 0.79 (6*e*^−10^)	0.43 ± 0.02 (4*e*^−5^)	1.36 ± 0.52 (4*e*^−10^)	0.69 ± 0.03 (8*e*^−24^)	72.69 ± 5.27 (1*e*^−8^)
MUNIT	17.86 ± 0.86 (4*e*^−11^)	0.44 ± 0.03 (3*e*^−4^)	0.91 ± 0.47 (9*e*^−8^)	0.73 ± 0.02 (2*e*^−14^)	71.42 ± 7.15 (8*e*^−8^)
DRIT++	17.62 ± 0.91 (8*e*^−12^)	0.42 ± 0.03 (4*e*^−6^)	0.92 ± 0.24 (2*e*^−13^)	0.71 ± 0.04 (9*e*^−15^)	76.26 ± 7.44 (5*e*^−6^)
DCLGAN	19.69 ± 0.99 (0.003)	0.47 ± 0.03 (0.062)	0.63 ± 0.16 (3*e*^−6^)	0.75 ± 0.01 (2*e*^−6^)	82.03 ± 6.45 (2*e*^−4^)
Chen et al.	18.53 ± 0.56 (8*e*^−10^)	0.44 ± 0.02 (3*e*^−4^)	1.65 ± 0.17 (1*e*^−20^)	0.73 ± 0.03 (2*e*^−14^)	80.15 ± 4.33 (6*e*^−8^)
Weakly MRUS	20.37 ± 0.92 (0.021)	0.49 ± 0.03 (0.12)	0.62 ± 0.19 (0.004)	0.76 ± 0.02 (0.001)	84.24 ± 2.32 (0.013)
Ours	**21.42** ± 0.43	**0.50** ± 0.03	**0.42** ± 0.15	**0.79** ± 0.01	**87.54** ± 3.12

**Table 2:** PSNR and SSIM on Region of Interest of TRUS images after rigid-body registration.

Method	PSNR ↑		SSIM ↑	
	Prostate	Rectum	Prostate	Rectum
CycleGAN [34]	16.808 ± 0.66 ††	18.609 ± 0.78 ††	0.400 ± 0.04 ††	0.407 ± 0.02 ††
ImgSA [13]	17.770 ± 0.58 ††	18.077 ± 0.98 ††	0.388 ± 0.03 ††	0.396 ± 0.02 ††
UNIT [21]	18.383 ± 0.44 ††	19.862 ± 0.73 ††	0.435 ± 0.04 ††	0.454 ± 0.02 ††
MUNIT [11]	17.751 ± 0.73 ††	19.41 ± 0.25 ††	0.445 ± 0.03 ††	0.441 ± 0.02 ††
DRIT++ [17]	18.519 ± 0.64 ††	18.770 ± 0.42 ††	0.458 ± 0.04 ††	0.467 ± 0.01 ††
DCLGAN [8]	17.671 ± 0.51 ††	20.253 ± 0.59 ††	0.478 ± 0.03 ††	0.434 ± 0.02 ††
Chen et al. [3]	18.756 ± 0.59 ††	19.117 ± 0.48 ††	0.429 ± 0.04 ††	0.431 ± 0.01 ††
Weakly MRUS [25]	20.05 ± 0.68 †	20.971 ±0.69 †	0.484 ± 0.04 ††	0.462 ± 0.02 ††
Ours	**21.208** ±0.53	**21.80** ±0.46	**0.492** ±0.03	**0.478** ±0.02

† and †† denote p-value < 0.05 and p-value < 0.01, respectively.

**Table 3:** Ablation study. There are five main components/mechanisms we introduce into our framework. We drop one of them each time and retrain the framework. ”Cycle breaking” means avoiding cycle consistency loss during training. We use the same as described in Table. 1.

Method	PSNR ↑	SSIM ↑	FID ↓	NCC ↑	DSC (%) ↑
w/o. cycle breaking	18.61 ± 0.44 ††	0.45 ± 0.01 ††	0.99 ± 0.11 ††	0.72 ± 0.04 ††	77.31 ± 5.52 ††
w/o. NCE network	17.74 ± 0.61 ††	0.44 ± 0.01 ††	0.57 ± 0.08 ††	0.75 ± 0.04 ††	77.76 ± 8.55 ††
w/o. normalization	18.16 ± 0.62 ††	0.46 ± 0.02 †	1.16 ± 0.64 ††	0.71 ± 0.02 ††	78.78 ± 7.49 ††
w/o. structure constraint	19.06 ± 0.78 †	0.43 ± 0.02 ††	0.53 ± 0.09 ††	0.73 ± 0.03 ††	81.20 ± 4.99 †
w/o. style adjustment	19.12 ± 1.07 †	0.48 ± 0.02	0.64 ± 0.13 ††	0.77 ± 0.04 ††	**87.84** ± 2.58
Ours	**20.42** ± 0.43	**0.50** ± 0.03	**0.42** ± 0.15	**0.79** ± 0.04	**87.54** ± 3.12

† and †† denote p-value < 0.05 and p-value < 0.01, respectively.

**Table 4:** PSNR and SSIM values for different edge detection thresholds.

Metric	200	250	300	350	400
PSNR	20.57	21.36	**21.42**	21.40	21.28
SSIM	0.498	**0.502**	0.501	0.493	0.488
